# Structural insights into human Kif7, a kinesin involved in Hedgehog signalling

**DOI:** 10.1107/S0907444911053042

**Published:** 2012-01-13

**Authors:** Marta Klejnot, Frank Kozielski

**Affiliations:** a The Beatson Institute for Cancer Research, Garscube Estate, Switchback Road, Glasgow G61 1BD, Scotland

**Keywords:** Kif7, Kif27, human kinesins, Hedgehog signalling

## Abstract

Kif7, a member of the kinesin 4 superfamily, is implicated in a variety of diseases including Joubert, hydrolethalus and acrocallosal syndromes. It is also involved in primary cilium formation and the Hedgehog signalling pathway and may play a role in cancer. Its activity is crucial for embryonic development. Kif7 and Kif27, a closely related kinesin in the same subfamily, are orthologues of the *Drosophila melano­gaster* kinesin-like protein Costal-2 (Cos2). In vertebrates, they work together to fulfil the role of the single Cos2 gene in *Drosophila.* Here, the high-resolution structure of the human Kif7 motor domain is reported and is compared with that of conventional kinesin, the founding member of the kinesin superfamily. These data are a first step towards structural characterization of a kinesin-4 family member and of this interesting molecular motor of medical significance.

## Abstract 2

The human Kif7 motor domain structure provides insights into a kinesin of medical significance.

## 1. Introduction

Members of the kinesin superfamily of motor proteins (Kifs) move unidirectionally along microtubules (MTs) in an ATP-dependent manner. They perform various functions and are best known for their role in neuronal transport (Hirokawa *et al.*, 2009 ▶) and mitosis/cytokinesis. Kinesins have been implicated in a range of diseases, usually related to their role in cellular transport, transport of pathogens and cell division (Mandelkow & Mandelkow, 2001 ▶).

**Figure 1:**
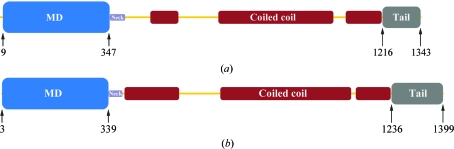
Bar diagrams of the human kinesin-4 family members (*a*) Kif7 and (*b*) Kif27. The globular motor domain (MD, blue) is followed by the short neck linker (Neck, violet), a stalk predicted to form a discontinuous coiled-coil region (red) and a C-terminal tail domain (grey).

Human Kif7 and Kif27, which are members of the kinesin-4 family (Katoh & Katoh, 2004*a* ▶,*b* ▶), are paralogues. They share 44% sequence identity overall and have even higher identity in the motor domain (61%). They possess an N-terminal globular motor domain that contains nucleotide-binding and MT-interacting regions, followed by a stalk domain predicted to form a discontinuous coiled coil and a globular C-terminal tail domain (Fig. 1 ▶).

Both proteins are orthologues of the *Drosophila melanogaster* kinesin-like protein called Costal-2 (Cos2) that plays a key role in Hedgehog (Hh) signalling. The Hedgehog ligands [Sonic hedgehog (SHh), Indian hedgehog (IHh) and Desert hedgehog (DHh)] are glycoproteins that play a crucial role during embryogenesis (Bijlsma *et al.*, 2004 ▶) and tumourigenesis (Magliano & Hebrok, 2003 ▶). Their signals are transduced through the transmembrane proteins Patched 1 (Ptch 1), Patched 2 (Ptch 2) and Smoothened (Smo). In the absence of Hh proteins, Ptch-family members suppress Smo activity, which becomes activated upon Hh ligand binding to the Ptch proteins. These events lead to the activation of the transcription factors Gli 1, Gli 2 and Gli 3. Direct interaction of the MT-­bound protein Cos2 with Cubitus interruptus (Ci) in the cytoplasm is believed to be essential for its sequential phosphorylation by three distinct kinases, PKA, GSKβ and CK1, and subsequent proteosome-dependent cleavage. The generated fragment, CiR, diffuses into the nucleus, where it co-represses Hh target genes (Tay *et al.*, 2004 ▶; Wang *et al.*, 2000 ▶). Human Kif7 and Kif27, which are putative ciliary motors with roles in the primary cilia, a sensory organelle (Verhey *et al.*, 2011 ▶), have been shown to interact with Gli transcription factors and are believed to fulfil distinct functions in regulating Gli proteins. Kif7, a critical regulator of mammalian Hh signalling, acts as both a positive and negative regulator in the Hh signalling pathway but performs this role differently from Cos2 in that it does not interact with Smo or Fu (Endoh-Yamagami *et al.*, 2009 ▶). The current hypothesis suggests that Kif7, as a plus-end-directed motor, transports Gli 2 away from the cilium, thus preventing its activation (Goetz & Anderson, 2010 ▶). In contrast, Kif27 interacts with Fu, which takes part in the construction of the primary cilium (Wilson *et al.*, 2009 ▶). Kif7 and Kif27 seem to work together in vertebrates to fulfil the role of the single Cos2 gene in *D. melanogaster*.

There are several reports describing Kif7 mutant alleles in mouse embryos and their characterization has shown the critical role of Kif7 in primary cilia formation and Hh signalling (Karel *et al.*, 2009 ▶; Cheung *et al.*, 2009 ▶; Endoh-Yamagami *et al.*, 2009 ▶). One of these alleles carried a Kif7_L130P_ mutation and caused death of mouse embryos at the end of gestation.

In humans, various Kif7 mutations have been linked to a range of neurodegenerative diseases, including Joubert (Dafinger *et al.*, 2011 ▶), hydrolethalus and acrocallosal syn­dromes (Putoux *et al.*, 2011 ▶). In addition, since inappro­priate activation of Hh signalling can lead to the formation of medulloblastomas, rhabdomyosarcomas, basal carcinomas and other tumour types (Goetz & Anderson, 2010 ▶) and given the role of Kif7 in human primary cilia formation and Hh signalling (Karel *et al.*, 2009 ▶), Kif7 might also be linked to other diseases such as cancer.

In our effort to characterize human kinesins of medical interest, we report here the crystal structure of the human Kif7 motor domain to 1.6 Å resolution. We compare this crystal structure with that of the founding member of the kinesin superfamily, conventional kinesin (kinesin-1 family). This is the first step towards the structural characterization of a kinesin-4 family member.

## 2. Materials and methods

### 2.1. Cloning, protein expression and purification

Codon-optimized cDNAs for Kif7_8–361_ and Kif27_1–341_ were purchased from GenScript and cloned into a modified pET-M20 *Escherichia coli* expression vector carrying an N-terminal Trx-fusion protein, a His tag and a TEV (tobacco etch virus) cleavage site. The sequences were verified by DNA sequencing. Subsequently, we cloned shorter con­structs using the following forward and reverse primers: Kif7_8–­347_, 5′-CGT GCC CAG TAA* ATC CGC AAC CGC GCC AC-3′; Kif27_1–339_, 5′-GCG AAC CGC GCG CGT TAG* ATT CTC GAG TAA TCG-3′; Kif27_6–341_, 5′-CT ATG GAA GCC ATG GCG GTG AAA GTT GCG-3′, 5′-GT GGT GCT CGA TTA CTC GAG AAT GTT ACG C-3′; Kif27_17–341_, 5′-GT ATT CGT CCC ATG GTG TGC AAA GAA GCG-3′, 5′-GT GGT GCT CGA TTA CTC GAG AAT GTT ACG C-3′. The Kif7 single point mutation (Kif7_8–347_L130P) was cloned employing the QuikChange Kit (Roche) with the following mutagenesis primer: 5′-C GAA GCC TTC AAA *CCG* ATC GAT GAA AAC GAC C-3′. All proteins were expressed in *E. coli* BL21 (DE3) pLysS expression cells (Novagen). Bacteria were grown in Terrific Broth (TB) medium (Sigma) at 310 K to an *A*_600_ of 0.7 and were subsequently induced with 0.5 m*M* isopropyl β-d-1-thiogalactopyranoside (IPTG; Sigma) at a temperature of 291 K. Overnight cultures were centrifuged (20 min, 277 K, 3300*g*) and resuspended in lysis buffer. Cells were lysed by sonication (eight cycles of 8 s on/off as 20 ml lysate fractions) and centrifuged (1 h, 277 K, 31 400*g*). Supernatants were pooled and the proteins were purified by affinity chromatography [1 ml HisTrap FF (GE Healthcare); buffer *A*, 50 m*M* piperazine-*N*,*N*′-bis(2-ethanesulfonic acid) (PIPES) pH 7.0, 250 m*M* NaCl, 2 m*M* MgCl_2_, 40 m*M* imidazole (Sigma); buffer *B*, 50 m*M* PIPES pH 7.0, 250 m*M* NaCl, 2 m*M* MgCl_2_, 500 m*M* imidazole], followed by overnight cleavage with TEV protease (0.05 mg TEV per milligram of protein). Prior to TEV cleavage, the buffer was exchanged to eliminate imidazole from the protein solution (HiPrep 26/10, GE Healthcare; 50 m*M* PIPES pH 7.0, 250 m*M* NaCl, 2 m*M* MgCl_2_). Subsequently, His-tagged Trx and TEV were removed by a second run on a His-trap affinity column. The final step of purification consisted of gel filtration (Superose S75; GE Healthcare) in gel-filtration buffer [50 m*M* PIPES pH 7.0, 250 m*M* NaCl, 2 m*M* MgCl_2_, 1 m*M* dithiothreitol (DTT; Sigma)].

### 2.2. Crystallization

All Kif7 and Kif27 constructs described above were used for crystallization trials at 10 mg ml^−1^ at 277 and 292 K in the presence and absence of 1 m*M* Mg^2+^ATP. The crystallization screens used were ComPAS, JCSG+, PACT (all from Qiagen), Ammonium Sulfate Grid Screen, Sodium Malonate Grid Screen, MPD Grid Screen, Crystal Screen HT, Crystal Screen Lite, PEG/Ion, PEG 6000 and PEG LiCl (all from Hampton Research). Kif7_8–347_ crystals grew in 20%(*w*/*v*) PEG 3350 in the presence of various salts (0.1 *M* magnesium formate dihydrate, 0.2 *M* lithium acetate, 0.2 *M* potassium sodium tartrate tetrahydrate, 0.1 *M* sodium acetate, 0.2 *M* ammonium nitrate, 0.2 *M* sodium nitrate). However, the best crystals were obtained in the presence of lithium acetate. All other protein constructs did not yield crystals under the conditions tested.

### 2.3. Analytical gel filtration

Analytical gel filtration was performed to determine the oligomeric state of the proteins (Superose 12 10/300 GL, GE Healthcare; buffer 50 m*M* PIPES pH 7.0, 250 m*M* NaCl, 2 m*M* MgCl_2_, 1 m*M* DTT). Experiments were conducted using a flow rate of 1 ml min^−1^ and an injection volume of 250 µl. Prior to running Kif7_8–347_ and Kif27_1–341_ constructs, the column was calibrated with proteins of known molecular mass (ribo­nuclease A, 16.7 kDa; carbonic anhydrase, 14.5 kDa; ovalbumin, 13.5 kDa; conalbumin, 13.0 kDa) and dextran blue. The *K*_av_ values were calculated for calibration proteins [(*V*_e_ − *V*_0_)/(*V*_c_ − *V*_0_), where *V*_e_ is the elution volume, *V*_0_ is the void volume and *V*_c_ is the column volume)] and plotted against the log of the molecular weights of the standards. The molecular masses of Kif7_8–347_ and Kif27_1–341_ were calculated from the resulting equation. The identities of the proteins were confirmed by mass spectrometry fingerprint analyses.

### 2.4. Data collection, structure determination and model refinement

Kif7_8–347_ crystals grown in 0.2 *M* lithium acetate, 20%(*w*/*v*) PEG 3350 on a microscale in hanging drops consisting of a 1:1 ratio of protein and reservoir solutions were transferred to cryoprotectant solution [20%(*w*/*v*) *meso*-erythritol (Sigma), 0.24 *M* lithium acetate and 24%(*w*/*v*) PEG 3350, 0.06 *M* Pipes, 0.3 *M* NaCl, 2.4 m*M* MgCl_2_, 1.2 m*M* DTT, 2.4 m*M* Mg^2+^ATP] and flash-cooled in liquid nitrogen. Data were collected on beamline I04-1 at Diamond Light Source and were processed with *XDS* (Kabsch, 2010 ▶) to 1.6 Å resolution in space group *P*2_1_2_1_2_1_. The structure was solved by molecular replacement with *MOLREP* (Vagin & Teplyakov, 2010 ▶) using the Kif3B structure (PDB entry 3b6v; Structural Genomics Consortium, unpublished work) as a search model. The asymmetric unit con­tained one copy of Kif7_8–347_ with bound Mg^2+^ADP positioned with *REFMAC*5 (Murshudov *et al.*, 2011 ▶) by rigid-body and restrained refinement. The model was subsequently improved with *Coot* (Emsley *et al.*, 2010 ▶) and further refined using *PHENIX* with TLS restraints (Zwart *et al.*, 2008 ▶).

### 2.5. Determination of protein concentrations

Protein concentrations were determined using the Beer–Lambert law with molar extinction coefficients (∊) of 10 430 mol cm^−1^ for Kif7_8–347_ and 18 910 mol cm^−1^ for Kif27_8–­347_, taking bound ADP into account (∊_ADP_ = 2500 mol cm^−1^), or by the Bradford method. Both methods yielded very similar protein concentrations, with differences of <10%.

### 2.6. Western blot

1 l cultures of native Kif7_8–347_ and of Kif7_8–347_L130P were grown, harvested and lysed as described previously. Subsequently, supernatants and pellets were analyzed separately. Supernatants were diluted tenfold. 200 µl of pellets was resuspended and boiled in 2 ml 6 *M* urea and diluted tenfold. SDS–PAGE was run with the following ratio: 10 µl sample, 8 µl loading buffer and 2 µl reducing agent. Proteins were then transferred onto nitrocellulose membrane (30 V, 400 mA, 1 h 20 min) in NuPAGE transfer buffer (Invitrogen) supplied with 20% methanol. The membrane was subsequently blocked overnight at 277 K using a solution of 5% milk powder in TBS-­T (Tris-buffered saline/Tween-20). The blocking solution was washed out with three cycles of 5 min washes with TBS-T and the membrane was incubated in 1% milk solution con­taining the 6×His mAb-HRP conjugate (Clontech) for 1 h at room temperature. After incubation, the excess conjugate was removed by three washes with TBS-T (5 min each) and a final rinse (5 min) in water. Finally, the membrane was developed with BM Chemiluminescence Western Blotting Substrate (Roche).

## 3. Results and discussion

**Figure 2:**
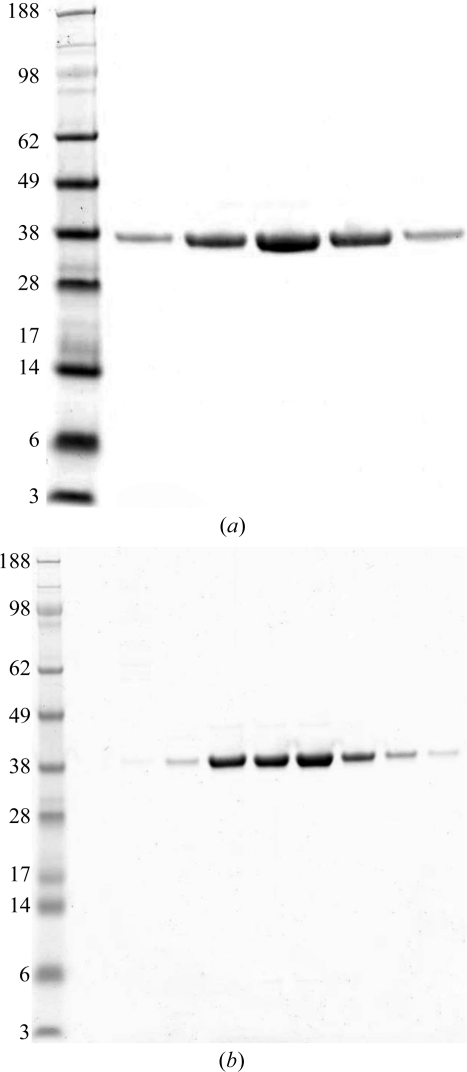
SDS–PAGE of gel-filtration profiles of (*a*) Kif7_8–347_ and (*b*) Kif27_1–341_. The left lane contains molecular-weight markers (labelled in kDa).

**Figure 3:**
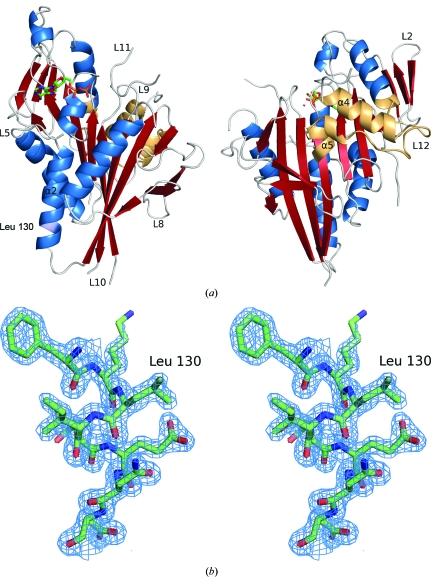
(*a*) Overall structure of Kif7_8–347_ in complex with Mg^2+^ADP (ball-and-stick model). α-Helices are shown in blue, β-strands are coloured red and loop regions are shaded in grey. The switch II cluster (helix α4/loop L12/helix α5) and the end of helix α6 preceding the neck linker region (not visible in this structure), which are thought to be pivotal for force generation in kinesins, are coloured orange. (*b*) Stereo-plot of helix α2 residues 128–132. The 2*F*_o_ − *F*_c_ map (coloured in blue) is contoured at 1σ. A single mutation in this region (L130P) causes embryonic lethality in mice.

Human Kif7 and Kif27 possess an N-terminal motor domain that contains nucleotide-binding and MT-interacting regions followed by a discontinuous α-helical region predicted to form a coiled coil responsible for oligomerization and a C-­terminal globular tail domain (Fig. 1 ▶). Mass spectrometry fingerprint analysis with coverage of 56 and 49% con­firmed that the recombinantly expressed and purified proteins were in fact Kif7 and Kif27. Analytical gel filtration showed that both constructs were monomeric (38 and 36 kDa), as expected for kinesin motor domains. Both proteins were >95% pure as judged by SDS–PAGE (Fig. 2 ▶). Of the seven Kif7 and Kif27 constructs used for crystallization, only Kif7_8–347_, which lacks the neck linker region, yielded crystals. Kif7_8–347_ crystallized as a monomer with one molecule per asymmetric unit. Data were collected to 1.6 Å resolution and the structure thus belongs to a small group of kinesins solved to a higher resolution. Data-collection and refinement statistics are summarized in Table 1 ▶. The final structure included residues 12–347 (missing regions: 107–114, 207–211, 230–241, 260–273) with bound Mg^2+^ADP (octahedrally co­ordinated) in the catalytic site and 328 water molecules. The refined structure contained 98.7% of residues in the most favourable region, 1.3% in allowed regions and no outliers. Fig. 3 ▶(*a*) presents the front and the back views of the overall structure of the Kif7 motor domain. Kif7 contains an eight-stranded β-sheet core with three major solvent-exposed α-helices on each side, displaying the characteristic arrowhead-like structure typical of all members of the kinesin superfamily solved to date (Kull *et al.*, 1996 ▶).

**Figure 4:**
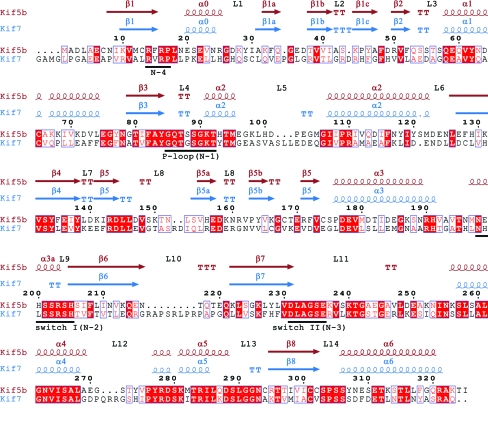
Sequence alignment and secondary-structure elements derived from the crystal structures of the Kif5b (conventional kinesin, kinesin-1; PDB entry 1bg2; Kull *et al.*, 1996 ▶) and Kif7 motor domains. The numbering relates to residues of Kif5b. Identical residues are coloured in white on a red background; similar residues are shaded in red. The ATP-binding pocket and the switch regions (N1–N4) are underlined in black.

Subsequently, we compared the Kif7 structure with that of conventional kinesin, the founding member of the kinesin superfamily. Alignment of Kif7_8–347_ with conventional kinesin (Kif5b) revealed their high structural similarity (Fig. 4 ▶). The sequence identity between the motor domains of Kif5b and Kif7 is 37%. There are two single insertions in the loop L2 region in the small three-stranded antiparallel β-sheet of Kif7. The P-loop or Walker motif (phosphate-binding region) is conserved in both proteins. In both structures, helix α2 is interrupted by loop L5, a highly important region for inhibitor binding in the mitotic kinesin Eg5. Kif7 contains a three-residue insertion in this loop. Although we could see some electron density for loop L5, residues Ala107–Glu114 are missing owing to its increased length and higher flexibility. In Kif7, the end of the second part of helix α2 has a single-residue deletion compared with Kif5b. Another two-residue insertion in Kif7 occurs in the loop L8 region, which is fully visible in the structure. Only residues Val159–Thr161 have an unclear density, which may suggest a dual conformation of this part of the loop. In Kif5b the switch I region (residues 199–205) consists of a small helix (α3a), whereas in Kif7 it forms a loop (L9). In Kif7 there is an eight-residue long insertion in loop L10; this region is disordered and is not visible in our structure. Switch II (Asp251–Glu257), which is highly conserved in both proteins and typically disordered in kinesins, is not fully visible in our Kif7_9–­349_ structure. The switch II cluster (helix α4/loop L12/helix α5) is in the so-called ‘down’ or ‘obstructive’ position and one would expect the neck linker (Arg350–Asn356) to be either unstructured or structured but in an undocked position with respect to the motor domain. However, the neck linker is not included in our protein owing to the short length of the Kif7 construct used for crystallization. Kif7_8–361_ contained the neck-linker region but did not crystallize, whereas Kif7_8–347_ lacking the neck linker revealed that the C-terminal residue Gln347 shows crystal contacts indicating that there would not be space for a neck linker in this crystal form. This might explain why the longer Kif7 construct did not yield crystals. The loop L11 region (Leu260–Ile273) is missing as in most other kinesin structures. Kif7 contains a three-residue insertion in loop L12 which is fully visible.

We also solved a mutated Kif7 motor-domain structure (PDB entry 2xt3). Despite four point mutations (Kif7_E226K_, Kif7_R268L_, Kif7_L269R_ and Kif7_H295N_), the overall structure of the motor domain remained unchanged (data not shown). In summary, the structure of human Kif7 resembles that of human conventional kinesin.

**Figure 5:**
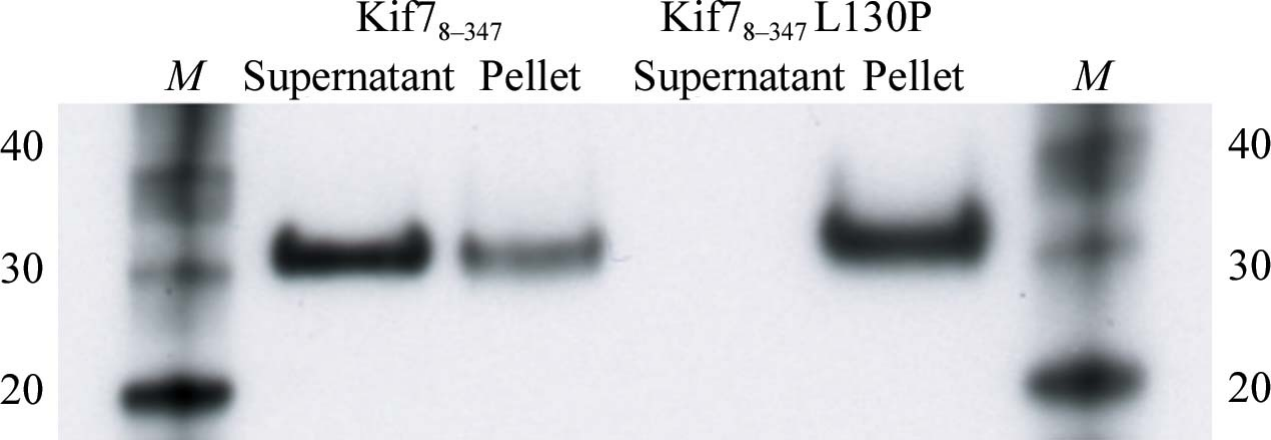
Western blot of soluble and insoluble fractions of Kif7_8–347_ and Kif7_8–347_L130P. Lanes *M* contain molecular-weight markers (labelled in kDa).

As previously mentioned, mutations in Kif7 alleles can disturb Hh signalling, leading to death of mouse embryos. For example, in mouse models a single mutation in the Kif7 motor domain (L130P) caused early embryonic lethality at the gestation stage. This mutation was introduced through *N*-­ethyl-*N*-nitrosourea induction in order to determine the impact on Hh signalling (Karel *et al.*, 2009 ▶). To understand how a single mutation in Kif7 can lead to such a dramatic event, we wanted to investigate the structure of the mutant. The construct carrying the mutation was expressed in *E. coli* but was insoluble (Fig. 5 ▶). Nevertheless, the structure of native Kif7 reveals that the L130P mutation would be situated at the end of the second half of the interrupted helix α2 (Fig. 3 ▶ *b*). We speculate that the mutation of leucine to proline, a known ‘helix breaker’, could cause a significant change to the secondary structure of the protein in this region. Another possibility could be that mutated Kif7 is degraded or aggregates *in vivo* owing to its misfolding. Also, there is one C—H—π interaction between Leu130 and Phe83 that is lost when leucine is mutated to proline.

In conclusion, we have pro­vided structural insights into the human Kif7 motor domain, a kinesin that is potentially implicated in several diseases. In the future, it will be interesting to determine whether Kif7 and its closely related paralogue Kif27 are proteins that are worth targeting in the Hedgehog signalling pathway (Sarangi *et al.*, 2009 ▶).

## Supplementary Material

PDB reference: Kif7, native, 4a14

PDB reference: mutant, 2xt3

## Figures and Tables

**Table 1:** Data-collection and refinement statistics for human Kif7_8–347_ Values in parentheses are for the highest resolution shell.

PDB entry	4a14
Beamline/detector	I04-1/Pilatus 2M
Resolution range (Å)	30–1.6
Space group	*P*2_1_2_1_2_1_
Unit-cell parameters (Å, °)	*a* = 45.99, *b* = 79.80, *c* = 95.05, α = β = γ = 90.0
Completeness (%)	99.9 (100)
*R*_merge_ (%)	3.2 (43.1)
Multiplicity	7.5 (7.5)
Mean *I*/σ(*I*)	30.1 (4.4)
No. of unique reflections	46998 (6746)
No. of copies per asymmetric unit	1
Refinement statistics	
*R*_work_/*R*_free_ (%)	18.25/20.67
No. of Mg^2+^ADP/waters	1/328
R.m.s.d. from ideal geometry	
Bond lengths (Å)	0.011
Bond angles (°)	1.547
Average *B* factors (Å^2^)	
Protein	27.6
Solvent	39.9
Ramachandran plot	
Residues in favoured regions (%)	98.7
Residues in allowed regions (%)	1.3
Outliers (%)	0.0
